# Faustovirus E12 Transcriptome Analysis Reveals Complex Splicing in Capsid Gene

**DOI:** 10.3389/fmicb.2018.02534

**Published:** 2018-10-23

**Authors:** Amina Cherif Louazani, Emeline Baptiste, Anthony Levasseur, Philippe Colson, Bernard La Scola

**Affiliations:** Assistance Publique – Hôpitaux de Marseille (AP-HM), Microbes, Evolution, Phylogeny and Infection (MEΦI), Institut Hospitalo-Universitaire (IHU) Méditerranée Infection, Institut de Recherche pour le Développement IRD 198, Aix-Marseille Université UM63, Marseille, France

**Keywords:** giant virus, faustovirus, transcriptome, capsid, splicing

## Abstract

Faustoviruses are the first giant viruses of amoebae isolated on *Vermamoeba vermiformis*. They are distantly related to African swine fever virus, the causative agent of lethal hemorrhagic fever in domestic pigs. Structural studies have shown the presence of a double protein layer encapsidating the double-stranded DNA genome of Faustovirus E12, the prototype strain. The major capsid protein (MCP) forming the external layer has been shown to be 645-amino acid-long. Unexpectedly, its encoding sequence has been found to be scattered along a 17 kbp-large genomic region. Using RNA-seq, we studied expression of Faustovirus E12 genes at nine time points over its entire replicative cycle. Paired-end 250 bp-long read sequencing on MiSeq instrument and double-round spliced alignment enabled the identification of 26 different splice-junctions. Reads corresponding to junctions represented 2% of mapped reads and mostly matched with the predicted MCP encoding sequences. Moreover, our study enabled describing a 1,939 bp-long transcript that corresponds to the MCP, delineating 13 exons. At least two types of introns coexist in the MCP gene: group I introns that can self-splice (*n* = 5) and spliceosome-like introns with non-canonical splice sites (*n* = 7). All splice-sites were non-canonical with five types of donor/acceptor splice-sites among which AA/TG was the most frequent association.

## Introduction

Faustoviruses are the first giant viruses of amoebae isolated using *Vermamoeba vermiformis* as cellular culture support (Reteno et al., 2015). Their capsids are icosahedral and virions are 200–240 nm large (Benamar et al., 2016). These viruses are distantly related to African swine fever virus, the causative agent of lethal hemorrhagic fever in domestic pigs (Alonso et al., 2018) and single species of family *Asfarviridae* (Iyer et al., 2001). In addition, two other faustovirus relatives have recently been described. Kaumoebavirus, also isolated on *V. vermiformis*, stands phylogenetically outside the asfarvirus–faustovirus group (Bajrai et al., 2016). Pacmanvirus, isolated on *Acanthamoeba castellanii*, is nested in phylogenetic analyses between asfarviruses and faustoviruses (Andreani et al., 2017). So far, 11 faustovirus isolates have been isolated, in all cases from sewage samples collected in France, Lebanon and Senegal (Cherif Louazani et al., 2017). Faustovirus-like sequences were also identified in metagenomes generated from arthropods as well as from febrile patients, healthy people, and from rodents (Temmam et al., 2015).

To better characterize the genomic diversity of faustoviruses, the genomes of the 11 isolates have been sequenced and annotated. These double-stranded DNA genomes contain between 456 and 491 kilobase pairs (kbp), have a G + C content comprised between 36.2 and 39.6%, and were predicted to encode between 457 and 519 genes (Benamar et al., 2016). Four lineages could be inferred from phylogenetic analyses of the core genome, with no clustering of the strains according to their geographical origin (Benamar et al., 2016; Cherif Louazani et al., 2017). For all these isolates, many hypothetical proteins were predicted, for which no function could be inferred due to the absence of recognizable homologs or conserved domains, their number being 148 among proteins encoded by the core genes.

In Faustovirus E12, the prototype virus of this group, proteomic analyses confirmed the presence in mature virions of 162 (33%) of the predicted proteins (Reteno et al., 2015). Moreover, cryo-electron microscopy showed the presence of a double protein layer encapsidating its genome (Klose et al., 2016). The major capsid protein (MCP) forming its external protein layer has been shown to be 645-amino acid-long. In addition, it folds into the double jelly roll motif that is characteristic of the capsid proteins of large nucleo-cytoplasmic double-stranded DNA viruses (NCLDV), a group of viral families that comprises the *Asfarviridae* family (Iyer et al., 2001). Strikingly, the sequences encoding the Faustovirus E12 MCP appeared to be scattered along a 17 kbp-large genomic region, with fragments located in both annotated and unannotated ORFs. This observation suggested that Faustovirus E12 uses an extended splicing during the expression of its MCP (Reteno et al., 2015; Klose et al., 2016).

*In silico* gene finding approaches have limitations in identifying genes, especially those that undergo post-transcriptional modifications or are present in the genomes of non-model organisms (Klasberg et al., 2016). The RNA-seq technology is particularly helpful in such cases. Using high throughput sequencing, RNA-seq allows high resolution identification of whole genome transcripts, of splicing events and splice junctions. It delineates the transcriptional structure of genes, and provides interesting information on gene expression levels and kinetics (Wang et al., 2009). Thus, previous studies of giant virus transcriptomes used RNA-seq to validate gene predictions and determine the precise 5′ and 3′ UTR structures of transcripts (Legendre et al., 2014, 2015). For Mimivirus, this approach increased the gene repertoire of 49 genes and detected a new component of the transcription apparatus (Legendre et al., 2011).

In the present study, we provide a comprehensive view of Faustovirus E12 genes expression through massive parallel sequencing of the total RNA-derived cDNA. We put a special focus on the identification of splicing events in the transcription process of the MCP encoding gene over the entire replicative cycle.

## Materials and Methods

**FIGURE 1:**
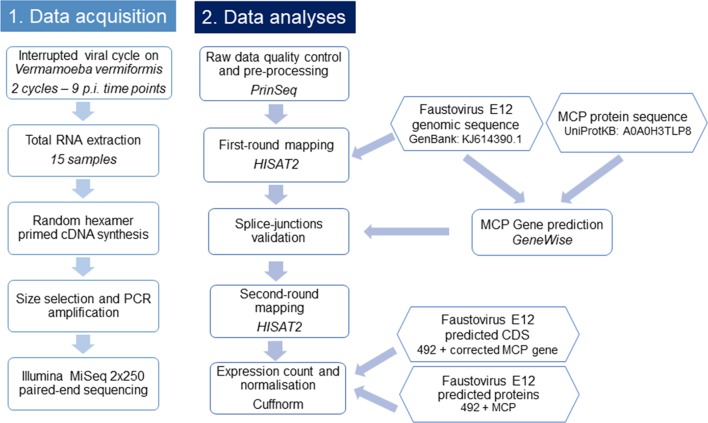
Flowchart illustrating the workflow of this study. This flowchart shows the general pipeline of this RNA-seq study, starting from sample preparation and RNA extraction to cDNA sequencing and data analyses. The biological interpretation of expression count was possible through the functional categories clustering of expressed genes.

A flowchart summarizing the main steps used in this study is presented in Figure 1.

### Data Acquisition

#### Virus Production and Infection Cycle

Faustovirus E12 was produced on *V. vermiformis* (strain CDC19) as in a previously described procedure (Reteno et al., 2015). Briefly, confluent monolayers of amoebae in Peptone-Yeast extract-Glucose (PYG) medium incubated at 28°C were rinsed with Page’s Amoeba Saline buffer (PAS) and centrifugated twice at 720 × *g* for 10 min, then put in a starvation medium at an adjusted concentration of 10^6^ cells/mL. The amoebae were then incubated at 30°C with a viral suspension at a MOI of five until complete cell lysis. The culture supernatant was then filtered at 0.45 μm to eliminate cellular debris and the filtrate was titrated by limited dilution assay.

For the interrupted infection cycle, adherent *V. vermiformis* incubated in PYG medium were put in contact with viral suspension at a MOI of 100. After incubation at 30°C for 1 h, the supernatant was removed, and the cultures were gently rinsed three times with PAS to eliminate excess virus. This marked time 0 (T0). For later time points, infected and rinsed amoebae were incubated at 30°C in PYG. Infected cells were pelleted by centrifugation at 720 × *g* for 10 min and were stored at -80°C in PBS.

In total, we realized two infection cycles with the following post-infection time points in duplicate: (*t* = 0, 15 min, 90 min, 3 h, 6 h, and 8 h), hereafter referred to as T0min-1, T15min-1, T90min-1, T3H-1, T6H-1, T8H-1 for cycle 1 and T0min-2, T15min-2, T90min-2, T3H-2, T6H-2, and T8H-2 for cycle 2. The second cycle included three additional late time points (*t* = 11 h, 17 h, and 20 h): T11H, T17H, and T20H.

### RNA Extraction and cDNA Sequencing

RNA was extracted using the RNeasy mini kit (Cat No: 74104, Qiagen, France) according to the manufacturer’s instructions. Total RNA was eluted in a 50 μL volume of RNase-free water. RNaseOUT (Thermo Fisher Scientific, France) was added to the elute to prevent RNA degradation. Genomic DNA contamination was checked using a PCR system targeting Faustovirus E12 DNA (forward primer: TCGGCATCAATCGCCTTATAG; reverse primer: GGCCAGAAGGGTCATTAACA). Two cycles of 30 min-DNase treatment using TURBO DNase (Invitrogen, France) incubation at 37°C were performed on the samples to achieve absence of DNA contamination. RNeasy MinElute Cleanup Kit (Qiagen) was used to purify DNA-free total RNA, using the manufacturer’s protocol with an RNA elution volume of 14 μL in RNase-free water.

The extracted total RNAs were reverse transcribed into cDNA using random primers with the SuperScript VILO Synthesis Kit (Invitrogen, France). cDNA amplicons were purified with the Agencourt AMPure XP system (Beckman Coulter Inc., CA, United States). Two sets of purified cDNA corresponding to the early and a complete Faustovirus E12 infection cycle were sequenced on a MiSeq instrument with the 2-bp × 250-bp paired-end strategy, using Nextera XT DNA sample prep kit (Illumina Inc., CA, United States). Quantified cDNAs were fragmented, tagged, then barcoded through limited cycle PCR amplification (12 cycles). After purification on Agencourt AMPure XP beads (Beckman Coulter Inc., CA, United States), the libraries were normalized on specific beads and pooled for sequencing. Each set was loaded on a separate flowcell.

### Data Analyses

#### Quality Control and Pre-processing of Reads

The raw data of paired-end reads were adapter trimmed. Adapter-free reads were checked for quality using PrinSeq web-version 0.20.1 (Schmieder and Edwards, 2011). Reads with over 10% Ns were filtered out. PolyA/T tails of over seven nucleotides (nt) were trimmed. Reads were quality trimmed from 5′-end with a sliding window of four and a step of three, with a mean Phred-scaled quality score cutoff of 20.

#### Study of Faustovirus Genes Expression

To identify potential splicing events in Faustovirus E12, we used a two-round alignment approach with a spliced-mapper: first, both pre-processed paired reads and singleton were mapped against the genomic sequence of Faustovirus E12 (GenBank accession no. KJ614390.1) using HISAT2 with minimum and maximum size of introns set to 20 and 5,000 bp (Kim et al., 2015). Spliced reads were extracted, and junctions manually validated using the Gene BED To Exon/Intron/Codon BED expander (Galaxy Version 1.0.0) (Afgan et al., 2016) and the Integrative Genomics Viewer (IGV) tool (Thorvaldsdottir et al., 2013). Junctions supported by at least two reads were included as known junctions in the second alignment round.

For each time point, reads mapping to the viral genes were quantified and the counts normalized with the geometric method using Cuffnorm (Galaxy Version 2.2.1.1) (Trapnell et al., 2010). For *t* = 0 to *t* = 8 h p.i., for which two biological replicates were available, both replicates were used as entries for a common normalized count (T0-c to T8H-c). To study the functional profile of the genes expressed during the replicative cycle, a BLASTp (Altschul et al., 1997) search of Faustovirus E12 annotated ORFs was performed against the Nucleo-Cytoplasmic Virus Orthologous Groups (NCVOGs) proteins database^1^ (Yutin et al., 2014). Hits with e-values below 1e-03 were considered significant and assigned to their corresponding NCVOGs. A weighted average of expressed genes in Fragments Per Kilobase of transcript per Million mapped reads (FPKM) was calculated for each functional category at each time point.

Proteins of the African swine fever virus (ASFV) identified in the purified particles (Alejo et al., 2018) were searched for homologs in Faustovirus E12 using BLASTp (Altschul et al., 1997) with 1e-03 as cutoff.

#### Study of the Major Capsid Protein Encoding Gene in Faustovirus E12

The 645-amino-acid protein sequence of the Faustovirus E12 MCP (UniProtKB accession no.: A0A0H3TLP8) was used to predict coding regions in the viral genome, using GeneWise (online version: wise2-4-1) (Li et al., 2015) with the GeneWise 623 algorithm, the flat null model and modeled splice sites as entry parameters. Predicted positions of exons were manually curated using information from junction reads. The coordinates of the corresponding junctions have been added to the file of known splice junctions for the second-round alignment of total RNA-derived cDNA.

## Results

### Faustovirus E12 Gene Expression

**FIGURE 2:**
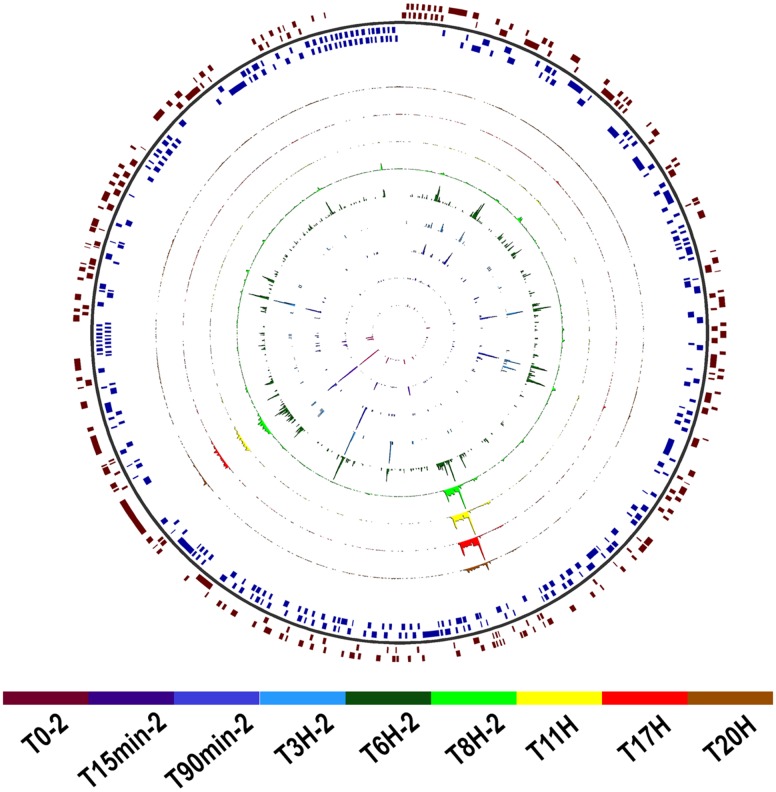
Map of Faustovirus E12 genome coverage during the replication cycle. The predicted protein coding sequences are represented on the external circle in *red* and *blue boxes* for the forward and reverse strand, respectively. The single base resolution coverage for each time point is reported in the colored concentric circles from *t* = 0 to 20 h for the complete replication cycle samples set. Position 0 is at the 12 o’clock position.

The transcriptome sequencing of Faustovirus E12-infected *V. vermiformis* resulted in 8,909,144 read pairs distributed over nine time points with two biological replicates corresponding to *t* = 0 min, 15 min, 90 min, 3 h, 6 h, and 8 h, and one replicate for *t* = 11, 17, and 20 h. After quality control, pre-processing and double-round mapping, reads corresponding to Faustovirus E12 represented <1% of the total number of generated reads, yet covering 93.5% of the genome positions with at least one read in at least one dataset. Single-base-resolution coverage maps across the genome for datasets of both cycles are reported in Figure 2 and Supplementary Figure 1. We observed a gradual increase in genome coverage during the replication cycle, illustrating an active transcription process starting early after infection. Two major shifts in coverage peaks profiles were observed after *t* = 90 min and *t* = 8 h, marking transitions from early to intermediate and from intermediate to late infection time points.

We detected that Faustovirus E12 expresses during its replicative cycle 90% (445/492) of its predicted genes including all but two genes that were assigned an NCVOG ID (116/118) (Supplementary Table 1). These two genes are a putative metal-dependent hydrolase (PRJ_Fausto_00294) and an uncharacterized protein (PRJ_Fausto_00234). Genes related to DNA replication, recombination and repair; nucleotide metabolism and transcription and RNA processing were expressed early and throughout the whole cycle. These include a hydrolase and a putative P-loop containing nucleoside triphosphate hydrolase, the ribonucleotide reductase small and large subunits and the hypothetical protein (PRJ_Fausto_00128) containing a Rho factor transcription termination domain.

**FIGURE 3:**
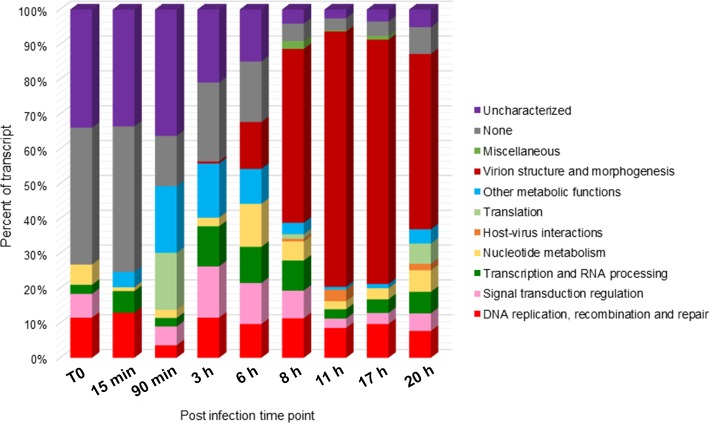
Functional categories distribution of expressed genes during the replication cycle of Faustovirus E12. Faustovirus E12 ORFs were assigned a functional category based on sequence homology with the Nucleo-Cytoplasmic Virus Orthologous Groups (NCVOG) proteins database. The ratio of expression per functional category is reported for each time point.

A large amount (32–75%) of the transcripts detected at early time points and up to 6 h p.i. corresponded to uncharacterized or poorly characterized proteins. Among the early expressed genes, we also found genes predicted to be involved in the ubiquitin-proteasome pathway and in host response regulation notably ankyrin repeats and membrane occupation and recognition nexus (MORN) repeat containing proteins. DNA directed RNA polymerase subunits are expressed starting from 90 min p.i. along with the transcription factor S-II (TFIIS), the mRNA capping enzyme and the translation initiation factor SUI1. The first transcripts corresponding to the MCP appeared at 3 h p.i while genes related to virion structure and morphogenesis were expressed starting from 6 h p.i. with increasing abundance in the late times. From 8 h p.i., the majority (50–73%) of the transcripts corresponded to proteins involved in virion structure and morphogenesis (Figure 3). Table 1 lists Faustovirus E12 genes predicted to encode for homolog proteins to those detected in ASFV purified particles proteome and their expression in late time points. All proteins forming the core shell in ASFV have their homologs in Faustovirus E12 expressed starting from 6 h p.i.: the 220 kDa polyprotein, the 62 kDa polyprotein and the protease necessary for their cleavage into their corresponding products. Other proteins found in the nucleoid of ASFV with their homolog predicted genes being expressed in Faustovirus E12 include all RNA polymerase subunits and RNA modification enzymes, transcription factors and DNA repair enzymes. Interestingly, using sequence homology, we were unable to identify in Faustovirus E12 genes predicted to encode for proteins detected in the outer and inner envelope of ASFV.

**Table 1:** ASFV virion-forming proteins with homologs detected in the transcriptome of Faustovirus E12.

ASFV gene	ASFV protein	Function	Faustovirus E12 homolog protein	T8H	T11H	T17H	T20H
B646L	Major capsid protein p72	Morphogenesis	MCP	+	+	+	+
CP2475L	Polyprotein pp220	Morphogenesis	AIB52024	+	+	+	+
CP530R	Polyprotein pp62	Morphogenesis	AIB52025	+	+	+	+
S273R	Polyprotein processing protease	Morphogenesis	AIB52094	+	+	+	+
O174L	DNA polymerase X	DNA integrity	AIB52077	+			
E296R	AP endonuclease	DNA integrity	AIB52085	+	+	+	+
E165R	dUTPase	DNA integrity	AIB51748	+	+	+	+
NP419L	DNA ligase	DNA integrity	AIB52048	+	+	+	+
NP1450L	RNA polymerase subunit 1	Transcription	AIB52040	+	+	+	+
EP1242L	RNA polymerase subunit 2	Transcription	AIB51752	+	+	+	+
H359L	RNA polymerase subunit 3-11	Transcription	AIB52132	+	+	+	+
D205R	RNA polymerase subunit 5	Transcription	AIB52137	+		+	+
C147L	RNA polymerase subunit 6	Transcription	AIB51823	+			
D339L	RNA polymerase subunit 7	Transcription	AIB52143	+	+	+	+
Q706L	VACV D11-like helicase	Transcription	AIB52129	+	+	+	+
B962L	VACV I8-like RNA helicase	Transcription	AIB51810	+	+	+	+
D1133L	VACV D6-like RNA helicase	Transcription	AIB52142	+	+	+	+
G1340L	VACV A7 early transcription factor large subunit-like	Transcription	AIB52005	+	+	+	+
NP868R	mRNA-capping enzyme	Transcription	AIB52055	+	+	+	+
C475L	Poly(A) polymerase	Transcription	AIB51816	+			
EP424R	Putative RNA methyltransferase	Transcription	AIB52114	+	+		+
EP152R	Protein EP152R		AIB51785				+
B169L	Uncharacterized protein		AIB51862	+			
H339R	α-NAC binding protein pH339R		AIB52116	+	+	+	+
M1249L	Uncharacterized protein		AIB51842	+	+	+	+
C129R	Uncharacterized protein		AIB51831	+			+
K421R	Uncharacterized protein		AIB51770	+		+	+
H240R	Uncharacterized protein		AIB52125	+		+	+
QP383R	Uncharacterized protein		AIB52104	+	+	+	+
C122R	Uncharacterized protein		AIB51826	+			
M448R	Uncharacterized protein		AIB51841	+	+	+	+

*The transcripts corresponding to the proteins of Faustovirus E12 with a homolog in African swine fever virus (ASFV) are indicated with a “+” when detected in the corresponding dataset*.

### Splicing Events in Faustovirus E12

**FIGURE 4:**
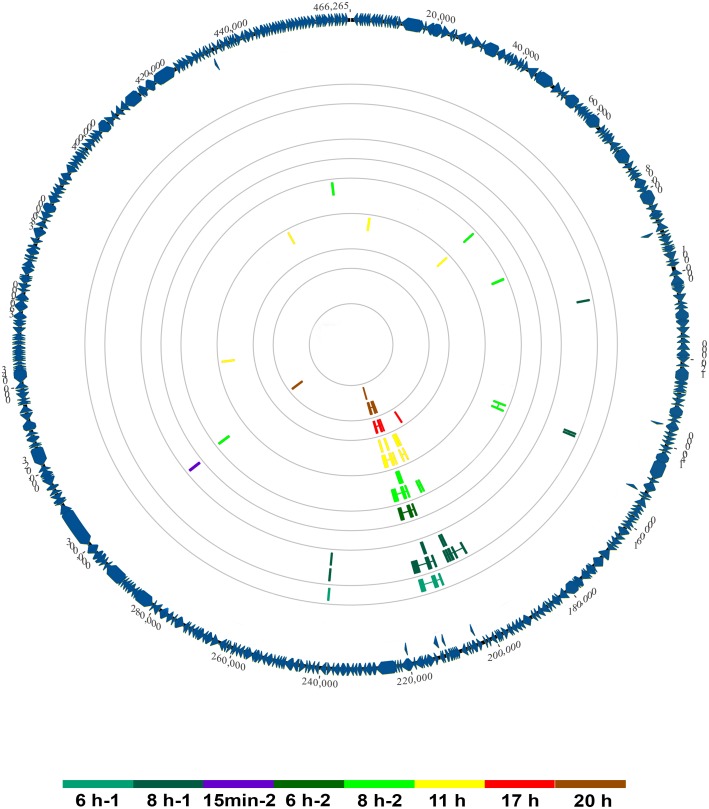
Genome-wide map of splicing events in Faustovirus E12 across its replication cycle. Predicted and curated splice junctions resulting from the second-round mapping of RNA-seq reads against the genome with HISAT2 are reported in *colored boxes* corresponding to the dataset where they were detected. When in close genomic coordinates, junctions appear in two layers for display purpose. The annotated protein coding sequences are represented on the external circle in *blue wedges*.

Using a splice-aware mapper and a double-round alignment strategy, with a manual validation of splice-junctions, we were able to identify 26 potential splice-junctions represented by at least two reads, with insert sizes reaching up to 3,256 bp. Figure 4 illustrates their distribution across the genome and throughout the replicative cycle of Faustovirus E12 in *Vermamoeba vermiformis*. We observed an uneven distribution of potential introns, with a high rate of splice-junctions grouped together in a single region of the genome and appearing in late times p.i. This region is the one predicted in previous studies to encode for the MCP of the virus (Klose et al., 2016). Overall, the number of junction-reads reached 2.7% (1,386) of the total mapped reads with 95.7% (1,326) of these reads aligning to the MCP encoding region.

### Faustovirus E12 Major Capsid Protein Transcription

In order to study the transcription of the MCP, we used both the gene prediction results of GeneWise, and the junction-reads after the first-round alignment. Junction-reads confirming the positions of predicted exons were added to the validated junctions file for the second-round alignment. The complete MCP transcript appears composed of 13 exons delineating 12 introns. Nine of the 13 exon–intron boundaries are supported by detected junction-reads. The mean intron length is 1,273 bp, with minimum and maximum lengths of 396 and 3,256 bp, respectively, and a mean G + C content of 35.2%. Exons forming the MCP coding transcript are significantly shorter (*p* = 0.0007, unpaired *t*-test) with length varying from 13 to 527 bp for a mean length of 149 bp and a mean G + C content of 43.9%. The exonic G + C content is significantly higher than that observed in introns (*p* < 0.0001, unpaired *t*-test).

An A/T substitution at transcript position 1,879 was found to generate a premature stop codon at protein position 631, suggesting the presence of a potential frame shift or post-transcriptional RNA editing mechanism.

Reads mapping to the MCP region represented 23.5% of the total mapped reads. The coverage of the intronic and exonic regions shifts during the replicative cycle. In early time points and until 3 h p.i, Faustovirus E12 appears to express transcripts corresponding to the intronic regions with coverage varying from 1.36 to 3.56, while for the same samples, the exonic regions have a null coverage. Starting from 6 h p.i., the exonic regions are detected and the highest coverage was observed in the sample T11H with 265.12 average coverage versus 16.89 in intronic regions of the same sample.

**FIGURE 5:**
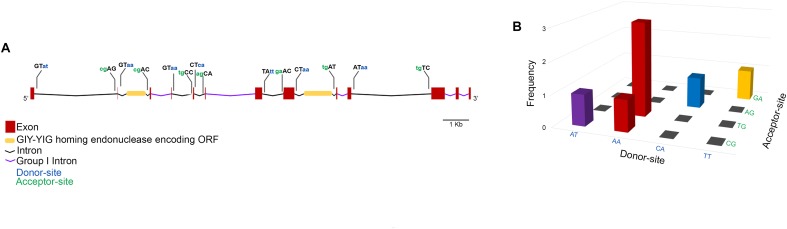
Faustovirus E12 major capsid protein gene structure. The MCP gene contains 13 exons and 12 introns among which five are group I self-spliced introns and two contain a GIY-YIG homing endonuclease inserted ORF. The donor and acceptor splice sites are represented for potentially spliceosome-processed introns **(A)**. The association of donor/acceptor splice site in these introns shows the high abundance of the donor site AA and its frequent association with TG acceptor site **(B)**.

To get a closer view on the mechanisms involved in the expression of the MCP gene, exon–intron boundaries were examined for conserved splice-sites that would suggest the presence of spliceosome-processed introns. Moreover, the intronic sequences were searched against the Rfam database for conserved motifs. Through this approach, five group I self-splicing introns were identified, and two introns were shown to contain an inserted ORF encoding a GIY-YIG homing endonuclease (Figure 5A). All the MCP gene exon-intron boundaries show non-canonical splice-sites with five types of donor-acceptor associations, among which AA/TG was the most represented (Figure 5B).

## Discussion

### An Overview of the Transcriptional Landscape of Faustovirus E12

This study represents the first exploration of the transcriptional landscape of Faustovirus E12. Using total RNA sequencing of infected cells at nine different time points covering the whole replicative cycle of the virus in *Vermamoeba vermiformis*, we were able to follow the temporal regulation of the viral transcription. Faustovirus E12 gene expression seems to follow the classical temporal regulation described in other giant viruses of amoebae and those of the former NCLDV group. Early on, transcripts related to the ubiquitin pathway were detected. This pathway has been described as a viral adaptation mechanism against host defenses. By transcribing its own components of the ubiquitin pathway, the virus can alter the host response to infection by modulating or degrading cell proteins (Iyer et al., 2006). Ankyrin repeats containing proteins are also expressed early and throughout the replicative cycle. In Poxviruses, these motif containing proteins have been described as modulators of host-range and their early expression could play a role in repressing host response by targeting the NF-κB pathway (Herbert et al., 2015). In parallel, to prepare its replication, the virus encodes the ribonucleotide reductase small and large subunits that provide the dNTPs necessary for viral DNA synthesis, therefore allowing virus growth in non-dividing cells (Gammon et al., 2010). Early mRNA transcripts are likely expressed using viral enzymes packaged within the infectious particles. Similarly to what is described in ASFV, the viral RNA polymerase is responsible for the transcription of all the viral genes but is expressed later during the replicative cycle (Rodríguez and Salas, 2013). Indeed, different RNA polymerase subunits, transcription factors and RNA modification enzymes are expressed late during the infection cycle, and likely translated into proteins incorporated to the virions during the assembly step. The comparison of the nucleoid components described by the proteomics analysis of ASFV particles and the late transcribed genes in Faustovirus E12 comforts this hypothesis (Alejo et al., 2018). Among the late transcribed gene products, we identified three enzymes homologous to the components of the base excision repair (BER) pathway described in ASFV. This pathway has been hypothesized to serve as an adaptation mechanism for viral replication in the cytoplasm of macrophages while not expressed in tissue cell cultures (Dixon et al., 2013). Formed by a DNA polymerase type X, a class II Apurinic/apyrimidinic (AP) endonuclease and a DNA ligase, all three detected in the transcriptome of Faustovirus E12 infected *Vermamoeba vermiformis*, this pathway could confirm the potential role of amoebae as training field for microorganisms’ resistance to macrophages (Greub and Raoult, 2004). The comparative study of Faustovirus E12 transcription on different host cells should be further investigated.

Faustovirus E12 DNA primase responsible for the initiation of DNA replication (AIB51821) in ASFV and the proliferating cell nuclear antigen-like protein that clamps the DNA polymerase to the DNA (AB52098) are expressed starting from 6 h p.i. with the onset of DNA replication (Dixon et al., 2013; Reteno et al., 2015). The most abundant transcripts detected in our study appear late during the infection cycle after *t* = 6h, at the viral factory step, and correspond to structural proteins responsible for the particles’ morphogenesis and packaging: the MCP, forming the external protein shell, is the most abundant transcript in late times. It is followed by the 220 kDa polyprotein and the 62 kDa polyprotein, both described as essential for the assembly of the core shell and the incorporation of the genomic DNA and nucleoid components in the mature virions (Andrés et al., 2002; Suárez et al., 2010). At *t* = 20 h p.i. as described in the developmental cycle of Faustovirus E12, most amoebae are lysed (Reteno et al., 2015) or appear at different stages of the replicative cycle of Faustovirus E12. This shows in our data with a mix of early and late transcribed genes in this dataset.

Although our data confirm the expression of most of Faustovirus E12 predicted protein-encoding genes, the low abundance of viral reads doesn’t allow further interpretation. With the high abundance of amoebal rRNA and mRNA in the total RNA extract, and in the absence of the *V. vermiformis* complete genome sequence from international sequence databases, the reads that could not be aligned against the Faustovirus E12 genome could not be unequivocally attributed to this amoeba. The possibility of using a ribodepletion strategy should be explored for future transcriptomic studies targeting giant viruses of amoebae.

### Corrected MCP Transcript

This study represents a first step forward in the understanding of the non-canonical splicing in Faustovirus E12 MCP expression. The use of paired-end 250 bp-long read sequencing on the MiSeq instrument allowed us to unambiguously identify splice junctions using a splice-aware mapper. Although HISAT2 is adapted to eukaryotic model organisms, the use of both prediction data and manual curation of the junction reads allowed us to describe a 1,939 bp-long transcript generated from a 17 kbp long gene and corresponding to the 645 amino acid-long sequence of the MCP forming the external protein shell of the mature Faustovirus E12 virions.

In early times of the replicative cycle, we observed transcription of regions corresponding to the introns of the MCP gene. Moreover, in the absence of RNA enrichment or selection step in our protocol, the observed transcribed introns in later times could be partially due to the presence of immature pre-mRNA particles in the total RNA extract.

Gene splicing was first described by two teams in Adenovirus 2 in 1977 (Berget et al., 1977; Chow et al., 1977). Subsequently, it has proved extensive in eukaryotes and as a central mechanism in gene regulation and protein diversity generation (Kelemen et al., 2013). The presence of introns has been suggested to increase gene expression by controlling the DNA accessibility or through the regulatory effect of some introns on the RNA polymerase (Hir et al., 2003). In Faustovirus E12, splicing was detected in the MCP gene, a high abundance protein encoding gene, with most spliced reads corresponding to this. This could reinforce the hypothesis that splicing plays a role in increasing gene expression (Alejo et al., 2018). However, the low abundance of viral reads in our datasets was a limit to the high confidence identification of other spliced genes.

Among giant viruses, introns were first described in the MCP encoding gene of *Acanthamoeba polyphaga mimivirus*, the firstly discovered giant virus of amoebae (Azza et al., 2009; Legendre et al., 2010). This gene was depicted as composed of three exons separated by two introns. A recent study compared MCP gene splicing profiles in *Mimiviridae* members from lineages A, B, and C and showed a lineage-independent variation in the structure and synteny of exons and intronic regions of this gene (Boratto et al., 2018). Introns were also detected in other conserved gene from giant viruses of amoebae, including those encoding DNA-dependent RNA polymerases and DNA polymerases (Yoosuf et al., 2012; Philippe et al., 2013; Deeg et al., 2018). Other NCLDV spliced genes include different genes of Paramecium bursaria chlorella virus 1 (PBCV-1) with 2 to 3 different types of introns described: spliceosome processed-like introns are present in the DNA polymerase and the pyrimidine dimer-specific glycosylase (PDG) genes and conserved in different chlorella viruses (Sun et al., 2000; Zhang et al., 2001). Group IB self-splicing introns are reported in a putative transcription factor TFII-like gene (ORF A125L) and in other regions of the viral genome where this intron propagated (Blanc et al., 2014).

In Faustovirus E12, a mixed mechanism may interfere with the expression of the MCP gene: the five group I introns could self-splice while the other exons use non-canonical splice-sites for their excision. The splice-sites, defined by the exon–intron boundaries in this virus are different from the usual canonical splice-sites observed in amoebae and eukaryotic cells, making it difficult to accurately identify them by using existing mapping programs alone. The use of known protein sequence to validate splice-junctions and a two-round alignment approach were beneficial for the definition of the MCP gene structure. The 13 exons forming this gene exhibit higher G + C content than their long flanking introns. This difference in G + C content could therefore play a role in the recognition of the exons by the splicing machinery, lowering the constraint on the intron-defined splice-sites, as hypothesized in higher eukaryotes (Amit et al., 2012).

Moreover, this Faustovirus E12 MCP gene exhibits inserted GIY-YIG homing endonuclease encoding ORFs in two different introns. The presence of this enzyme has been thought to play a role in host competition among related viruses, impeding virus replication by cleaving genes essential to virus replication and contributing to the creation of chimeric genomic regions containing parasitic genetic elements in these genomes (Deeg et al., 2018).

As a summary, Faustovirus E12 MCP splicing presents three main features that make it unusual: (i) The number of introns: although splicing has been described in other viruses, the number of introns is generally limited to 1–3 introns. It is, to our knowledge, the first description of a spliced gene composed of 12 introns in a virus. (ii) The size of introns: with a mean length of 1,273 bp, the introns forming the MCP gene of Faustovirus E12 are larger than previously described introns in viruses. The gene structure with multiple large introns is otherwise common in cellular organisms. (iii) The mixed mechanisms that could be in play in the splicing of the MCP gene: Faustovirus E12 MCP gene is formed both of group I introns, and potential spliceosomal introns. Moreover, the potentially spliceosomal introns use non-canonical splice-sites in their excision. Overall, the complexity and unusual splicing observed in Faustovirus E12 contribute to blurring the border between giant viruses of amoebae and cellular organisms, and thus strengthen the delineation of these viruses as different complex entities compared to classical viruses.

## Data Availability

The datasets generated for this study were submitted to the European Nucleotide Archive database and are available under the accession numbers ERR2724024 to ERR2724038.

## Author Contributions

ACL and BLS conceived and designed the experiments. AL, PC, and EB contributed to materials and analysis tools. ACL, AL, PC, and EB analyzed the data. ACL, AL, PC, and BLS wrote the paper.

## Conflict of Interest Statement

The authors declare that the research was conducted in the absence of any commercial or financial relationships that could be construed as a potential conflict of interest.

## Footnotes

1 ftp://ftp.ncbi.nih.gov/pub/wolf/COGs/NCVOG/
