# Supplementary material for: Faustovirus E12 Transcriptome Analysis Reveals Complex Splicing in Capsid Gene
Source: Front Microbiol. 2018 Oct 23;9:2534. doi: 10.3389/fmicb.2018.02534 (PMC6247863; doi:10.3389/fmicb.2018.02534)
Supplement: Supplementary file 2 [file Data_Sheet_1.pdf]

Faustovirus E12 transcriptome analysis reveals complex splicing in capsid gene

Amina CHERIF LOUAZANI, Emeline BAPTISTE, Anthony LEVASSEUR, Philippe COLSON, Bernard LA SCOLA*

***Correspondence:** Bernard La Scola, MD, PhD, Pôle des Maladies Infectieuses, Aix-Marseille Université, IRD, Assistance Publique – Hôpitaux de Marseille (AP-HM), Microbes, Evolution, Phylogeny and Infection (MEΦI), Institut Hospitalo-Universitaire (IHU) - Méditerranée Infection, France, 19-21 Boulevard Jean Moulin, 13385 Marseille Cedex 05, France. Telephone: +33 4 91 32 43 75, Fax: +33 4 91 38 77 72, E-mail: bernard.la-scola@univ-amu.fr

1 Supplementary Figures and Tables

1.1 Table 1. Expression Data of Faustovirus E12 thoughout its replicative cycle

Normalized expression count of Faustovirus E12 genes and their NCVOG categories distribution. Expression counts are calculated in Fragments Per Kilobase Million (FPKM) for each post infection time point using both replicates data when available (from T0 to T8H).

1.2 Figure 1. Genome-wide coverage map of Faustovirus E12 from T0 to T8H

The coverage at each position and each time point of the first samplesets (T0 to T8H) is reported in the concentric circles starting at 12 o'clock position for base 0. The genome predicted protein coding genes are represented on the external circle in red and blue boxes for forward and reverse strand, respectively.

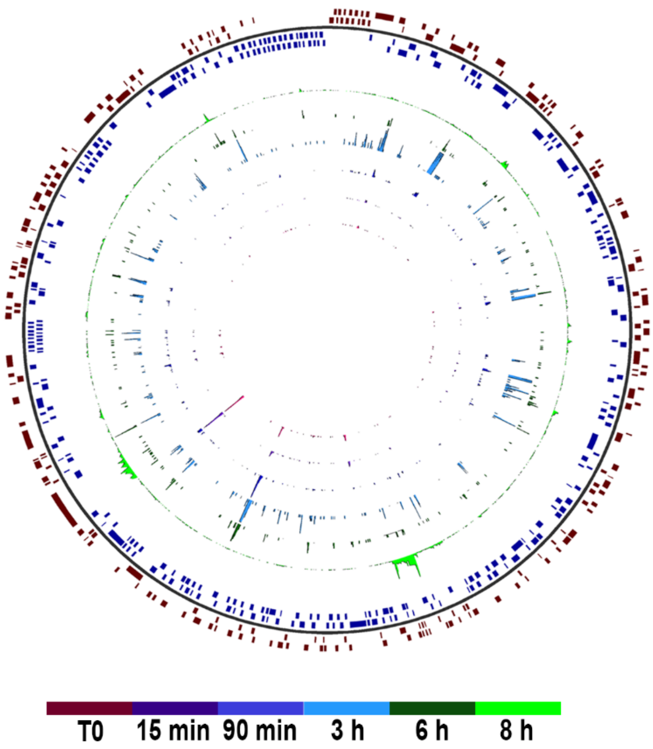
T0
15 min
90 min
3 h
6 h
8 h
